# The Use of Virtual Care in Patients with Hematologic Malignancies: A Scoping Review

**DOI:** 10.3390/curroncol29020076

**Published:** 2022-02-07

**Authors:** Adam Suleman, Abi Vijenthira, Alejandro Berlin, Anca Prica, Danielle Rodin

**Affiliations:** 1 Faculty of Medicine, University of Toronto, Toronto, ON M5S 1A1, Canada; 2 Division of Medical Oncology and Hematology, Princess Margaret Cancer Centre, Toronto, ON M5G 2C1, Canada; abi.vijenthira@uhn.ca (A.V.); anca.prica@uhn.ca (A.P.); 3 Radiation Medicine Program, Princess Margaret Cancer Centre, Toronto, ON M5G 2C1, Canada; alejandro.berlin@rmp.uhn.ca (A.B.); danielle.rodin@rmp.uhn.ca (D.R.); 4 Department of Radiation Oncology, University of Toronto, Toronto, ON M5S 1A1, Canada

**Keywords:** virtual care, telemedicine, hematologic malignancies, cancer

## Abstract

There is increasing interest from cancer patients and their healthcare providers in the use of virtual care in routine clinical practice. In the setting of hematologic malignancy, where patients often undergo complex and immunodepleting treatments, understanding how to use virtual care safely and effectively is critically important. We aimed to describe the use of virtual care in patients with hematologic malignancies and to examine physician- and patient-reported outcomes in the form of a systematic scoping review. An electronic search of PubMed, Ovid MEDLINE, Elsevier Embase, Scopus, and EBSCO CINAHL was conducted from January 2000 to April 2021. A comprehensive search strategy was used to identify relevant articles, and data were extracted to assess the study design, population, setting, patient characteristics, virtual care platform, and study results. Studies were included if they described the use of virtual care for patients with hematologic malignancies; commentaries were excluded. Fifteen studies met the inclusion criteria after abstract and full-text review. Three studies found that app-based tools were effective in monitoring patient symptoms and triggering alerts for more urgent follow-up. Four studies described the use of phone-based interventions. Five studies found that videoconferencing, with both physicians and oncology nurses, was highly rated by patients. Emerging themes included high levels of patient satisfaction across all domains of virtual care. Provider satisfaction scores were rated lower than patient scores, with concerns about technical issues leading to challenges with virtual care. Four studies found that virtual care allowed providers to promptly respond to patient concerns, especially when patients were experiencing side-effects or had questions about their treatment. Overall, the use of virtual care in patients with hematologic malignancies appears feasible, and resulted in high patient satisfaction. Further research is needed in order to evaluate the optimal method of integrating virtual care into clinical practice.

## 1. Introduction

The COVID-19 pandemic has motivated clinicians to re-examine the way healthcare is delivered. Many physicians have transitioned to virtual care, most often in the form of video or phone appointments [1,2], in order to provide adequate care for patients while minimizing avoidable contact and the risks of infection [3]. Patients with hematologic malignancies have been particularly vulnerable during this period due to their underlying malignancy and immunodepleting treatments [4,5]. The use of virtual care also provides a mechanism to overcome challenges associated with travel time and distance to the hospital, which have previously been identified as barriers to access in this population [6]. This transition has motivated a discussion amongst patients and providers as to how virtual care can and should be routinely used in the management of patients with hematologic malignancies.

Patients and physicians have had mixed opinions on the value, safety, and feasibility of virtual care [7,8,9]. A 2020 study at a large comprehensive cancer center found that a majority of patients (68%) with virtual care visits were highly satisfied, but over one-third of physicians felt that virtual care early in the COVID-19 pandemic compromised quality [10]; similar findings have been observed in other jurisdictions [9]. Amongst providers, physical examination to identify disease recurrence or treatment complications, along with the desire to form a personal connection with patients, have been used as important justifications for in-person visits [11,12]. Among patients, however, a study of 1077 patients in a radiation oncology clinic who received telemedicine consultations between December 2019 and June 2020 found no significant differences between office and telemedicine consultations in terms of overall satisfaction, the quality of the physician’s explanations, or the levels of physician concern and friendliness [13]. Furthermore, Hsiehchen et al. found that virtual care for patients with solid tumor malignancies saved an average of 211.4 min of travel time per patient and resulted in no change in rates of chemotherapy discontinuation, emergency department visits, or admissions [14].

The incorporation of virtual care into routine practice was spearheaded by the pressing needs early in the COVID-19 pandemic and the associated public health measures to contain the spread of the virus, but its ongoing use suggests that it may remain permanently integrated into cancer care delivery [15,16]. However, the majority of research on virtual care has focused on patients with solid tumors, and there are few published data on virtual care in hematologic malignancies. Given the clinical spectrum of hematologic malignancies, there may be a wide variety of patients who can appropriately and safely receive high-quality virtual care, which may be satisfying to both patients and providers. However, given the aggressive nature of many hematologic malignancies, and the use of treatments that cause significant immunosuppression and serious complications, the appropriateness and safety of virtual care replacing in-person visits needs to be examined. As virtual care use increases, it is important to understand how it can be safely and appropriately integrated into the care of patients with hematologic malignancies in order to enhance care delivery.

The aim of this scoping review is to further our understanding of how virtual care is being used for patients with hematologic malignancies, the experience of both patients and providers, and the impact of its use on patient outcomes. We also aimed to consider the challenges associated with virtual care delivery, and how virtual care can be optimally and safely integrated into routine practice.

## 2. Materials and Methods

### 2.1. Methodology of Scoping Review

We carried out a structured scoping review of the literature as per PRISMA guidelines in order to determine how virtual care is being used in the delivery of care for patients with hematologic malignancies, and to identify areas for future study. As an emerging area of study, the methodology of a scoping review was selected to provide an overview of the state of the literature, and to include a broad range of evidence about the use of various methods of virtual care in the hematologic malignancy population [17].

### 2.2. Data Sources and Search Terms

Databases searched included PubMed (January 2000 to April 2021), Ovid MEDLINE (2000 to April 2021), Elsevier Embase (2000 to April 2021), Scopus (2000 to April 2021), and EBSCO CINAHL (2000 to April 2021). A start year of 2000 was selected for all databases because virtual care prior to the year 2000 was felt to be less representative of the way virtual care can be delivered with contemporary technologies. A variety of search terms to encompass hematologic malignancies were used, including hematologic neoplasms, leukemia, lymphoma, myeloma, and stem cell transplant. Search terms to encompass virtual care included telemedicine, telehealth, eHealth, mHealth, and virtual care. Our full search strategy can be seen in Methodology S1.

### 2.3. Article Selection and Data Abstraction

Articles were included if they focused on patients with any hematologic malignancies during active surveillance, treatment, or post-treatment follow-up. Articles were included if they were peer reviewed and reported on a virtual care modality that involved direct clinician–patient interaction, including app-based, phone communication, or video conference. There was no minimum number of patients with hematologic malignancies described in the articles required for inclusion, but all included texts were required to specify the hematologic malignancy population in the study. For the purpose of this review, articles were limited to the year 2000 onwards and the English language. Articles were excluded if they were commentaries or editorials without any specific intervention or outcome evaluation. Titles and abstracts were screened by A.S. and A.V. Full-text articles were reviewed by two independent reviewers (A.S. and A.V.), with any disagreements resolved by discussion with two additional reviewers (D.R. and A.P.). Data were extracted to assess the study design, population, setting, patient characteristics, method of virtual care, and study results. Articles were coded based on the method of virtual care delivery, study population, and outcomes assessed. Coding of the key themes in the article was initially performed by A.S. The coding approach was then reviewed and discussed by all authors (A.V., A.B., A.P., D.R.), and the core areas of focus were agreed upon. 

## 3. Results

**Figure 1:**
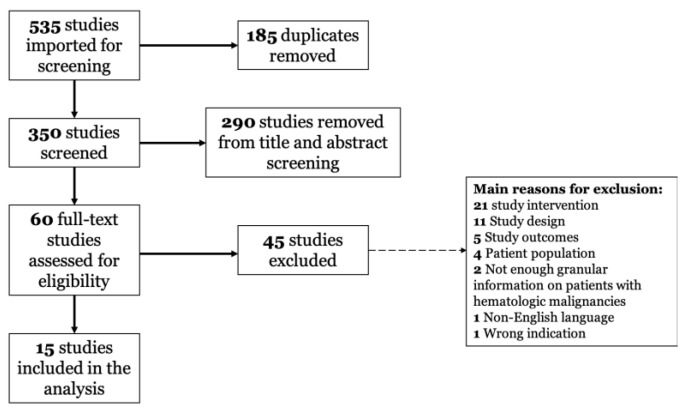
PRISMA diagram of studies assessed for eligibility of inclusion in the study.

A flow diagram of article inclusion in this review is shown in Figure 1. Our search returned 535 records; after removal of duplicates, 350 unique records remained; of these, 290 studies failed to meet the inclusion criteria or met one or more exclusion criteria based on title and abstract screening. Full-text review of the 60 remaining articles was performed, and 45 studies were excluded. Finally, 15 articles were included in the review: 13 studies were retrospective, with 1 report on a cohort of patients from a randomized trial [18], and 1 prospective pilot study [19].

### 3.1. Publication Demographics

**Figure 2:**
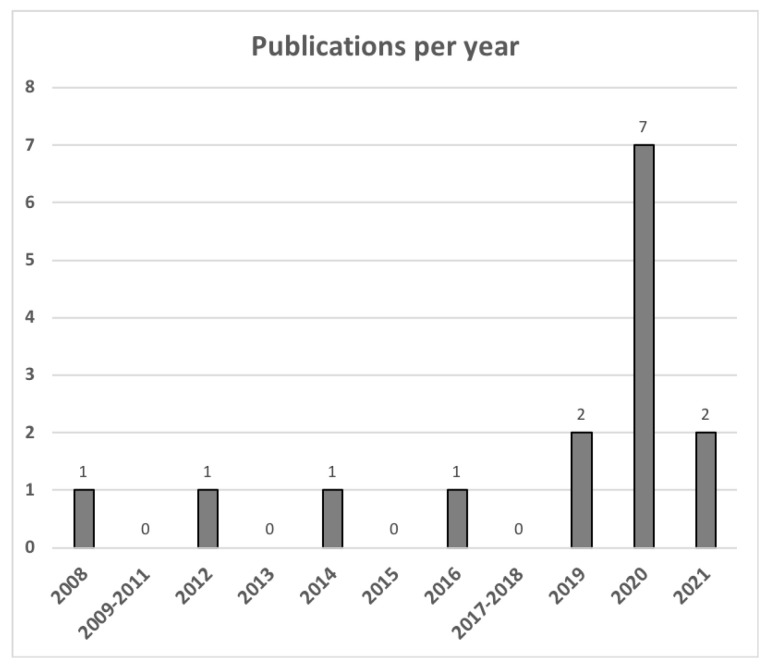
Trend of publication frequency of articles included in the scoping review.

Articles included in this review reported on the use of virtual care in patients with lymphoma (*n* = 4), allogeneic and autologous stem cell transplant (*n* = 4), chronic myeloid leukemia (*n* = 3), acute leukemia (*n* = 1), and combinations of the above (*n* = 3). Figure 2 shows a trend in publications over time, with many studies published during the COVID-19 pandemic (i.e., March 2020 onwards). A description of the characteristics of the included articles is shown in Table S1. A majority of the published studies included patients from North America (*n* = 7) and Europe (*n* = 5). Additional studies included populations from Australia, China, and Nepal. Virtual care modalities assessed included app-based care (*n* = 3), phone (*n* = 4), video (*n* = 5), and mixed intervention (*n* = 2). Articles described patients on active treatment (*n* = 9) and ongoing surveillance after therapy (*n* = 6).

### 3.2. Use of App-Based Interventions

Three articles described the use of virtual app-based interventions for care of patients with hematologic malignancies [20,21,22]. These interventions were used to monitor symptoms in patients with a variety of hematologic malignancies, and to facilitate timely communication between patients and their treating physicians. Ector et al. assessed the use of a web-based intervention and mobile app (CMyLife) for patients with chronic myelogenous leukemia (CML) [20]. Eleven patients were recruited via national patient associations, and focus groups with the patients and thirteen hematologists were conducted after the pilot test app was released. The web-based intervention provided information about CML, but also allowed input of symptoms, leading to screen consultations through the app with the treating hematologists. Zhang et al. described the use of an app for post-treatment survivorship after treatment for lymphoma [22]. The app allowed for both scheduled tasks and requests to be sent, and for alerts to be triggered based on patient-reported side-effects. Oncologists were then able to speak with patients based on the alerts. The app was used by 856 patients; 218 patients had 616 symptom-triggered alerts that resulted in follow-up with their oncologist. Finally, Poudyal et al. described the use of the Viber app for communication with patients being treated for acute leukemia and other hematologic malignancies during the COVID-19 pandemic in Nepal [21]. Patients were able to connect easily with their hematologists through the app to access documentation of their care. During the time when travel was banned due to the pandemic, app-generated documentation was used to obtain permission for travel to and from the hospital. The main limitation described in these studies was physicians’ concerns about the additional time required to respond to all app-based triggers.

### 3.3. Use of Phone Call Interventions

Four articles described the use of phone-based interventions in the care of patients with hematologic malignancies [18,23,24,25]. One retrospective study by Rueter et al. assessed the use of a telemedicine phone call by a coordinating nurse to proactively track symptoms related to therapy for patients with diffuse large B-cell lymphoma being treated with R-CHOP chemoimmunotherapy from November 2006 to June 2011 [23]. These calls could trigger further assessments or provide assistance on how to manage symptoms. Of the 418 patients included in this study, 34.2% of them had virtual care support through phone call assessments. 

Condom et al. described the changes in visits for patients with various hematologic malignancies in Barcelona from 13 March to 12 April 2020 during the initial phases of the COVID-19 pandemic [24]; the number of phone visits increased by 761%, with a 70% decrease in in-person follow-up visits. Similarly, Lupo-Stanghellini et al. described use phone teleconsults in Northern Italy from 24 February to 31 March 2020 for patients with a history of allogeneic stem cell transplant [25]; 29 out of 50 patients with chronic graft-versus-host disease had visits conducted by phone, and 125 out of 203 visits for non-respiratory related illnesses were conducted virtually.

One phone-based intervention study by Applebaum et al. reported on a cohort of patients included in a randomized controlled trial who had post-traumatic stress disorder after hematopoietic stem cell transplant [18]. Forty-six patients were randomized to a single phone assessment as the standard of care or a phone assessment with phone-administered cognitive behavioral therapy (CBT). The use of phone-based CBT was found to decrease general distress over time and increase therapeutic alliance.

### 3.4. Use of Video Interventions

Five articles described the use of video for the delivery of virtual care to patients with hematologic malignancies [19,26,27,28,29]. One study of patients in rural Australia who had undergone an allogeneic bone marrow transplant found that 328 out of 441 (74%) of patients preferred follow-up at a satellite clinic with video teleconferencing [26]. Another group from the United States found that patients with chronic lymphocytic leukemia had improved understanding of their disease after video consultation, and the majority would recommend video conferencing [27,28]. Overend et al. found that for 45 patients with chronic hematologic malignancies (mainly CLL and follicular lymphoma) in Canada, teleclinics by an oncology nurse were preferred by 62% of patients [29].

### 3.5. Use of Mixed Interventions

Two papers described a combination of phone and video for the management of patients with hematologic malignancies [30,31]; both described the transition to virtual care during the COVID-19 pandemic, with many visits conducted virtually early on during the pandemic. Fattizzo et al. described approximately half of visits being conducted via telemedicine by the third week of the pandemic [31]. The specific method of virtual care delivery was not described in either study, and there were no descriptions comparing methods of virtual care. 

### 3.6. Virtual Care during the COVID-19 Pandemic

Four studies specifically discussed the use of virtual care during the COVID-19 pandemic [24,25,30,31]; in all of them, the number of telemedicine visits, by either phone or video, significantly increased during the initial phases of the COVID-19 pandemic. One study that looked at patients with a variety of malignancies, including both solid tumor and hematologic malignancies, found that new patients had less anxiety, fatigue, and concerns of financial distress when care was performed virtually [30]. The uptake of virtual care was most often described in the time period of March–April 2020.

### 3.7. Satisfaction with Virtual Care

#### 3.7.1. Patient Satisfaction

Six articles (four using video conferencing, one app-based, and one mixed-methods study) specifically assessed components of patient satisfaction with virtual care [19,20,26,28,29,30]; satisfaction was assessed using patient survey data in all cases. There was congruence between studies of patients reporting less financial distress when care was performed virtually, including patients with a variety of hematologic malignancies and after allogeneic bone marrow transplant [26,30]. Nawas et al. found that out of 27 patients after hematopoietic stem cell transplant who had virtual visits during both their inpatient and outpatient visits, overall satisfaction with telehealth visits was rated 4.12 out of 5 [19]. On a scale of 1 to 5, with 1 representing a strong disagreement with the statement, an average rating of 1.93 out of 5 was found when patients were asked if they felt they would have received better care with an in-person visit, suggesting that patients were highly satisfied with virtual care [19]. Overend et al. also found that amongst 53 patients with indolent lymphoma, 62% preferred follow-up through a telehealth clinic when given the option [29]. 

#### 3.7.2. Provider Satisfaction

Two studies addressed provider satisfaction with virtual care using surveys [19,20]. In both app-based and video-based interventions, treating physicians were most often concerned about technical issues impairing their perceived ability to seamlessly provide care, as well as the additional time required to respond to patient messages. Satisfaction scores were rated lower for providers compared to patients [19].

### 3.8. Clinical Outcomes with Virtual Care

Seven studies assessed specific outcome data when virtual care was performed for patients with hematologic malignancies [18,21,22,23,27,30,32]. One emerging theme was the improved ability for the treating physician to understand patients’ symptoms and address them promptly and appropriately through virtual care [18,27,30]. Rueter et al. retrospectively reviewed 418 patients with a diagnosis of diffuse large B-cell lymphoma on treatment from November 2006 to June 2011 and compared all-cause mortality for patients who had telemedicine phone call follow-ups with a coordinating nurse to that for patients who did not have telemedicine visits [23]. The use of proactive telemedicine visits resulted in a 40% decrease in mortality risk compared to patients who had no telemedicine visits (HR 0.6, 95% CI 0.4–0.9). The study was not randomized, but suggests that telemedicine during active therapy may provide a mortality benefit [23].

Poudyal et al. described how using an app in Nepal during the COVID-19 pandemic improved communication with patients and, when travel was restricted by law, allowed all patients to travel to the hospital to attend their appointments by showing their communication with their treating physicians to authorities [21]. As a result, in that specific context, no patients had delays in chemotherapy. Runge et al. found that for patients with lymphoma, only 7 out of 128 patients had dose reductions in their regimens based on physical exam findings alone, suggesting that in-person visits seldom influence clinical decisions during active treatment [32].

## 4. Discussion

This scoping review is the first synthesis of the literature on virtual care in patients with hematologic malignancies. Patients with hematologic malignancies represent a unique group of patients who are often exposed to high-intensity chemotherapy regimens with a high risk of side-effects, and who often remain at risk of various toxicities and complications [33]. Our findings suggest that implementing strategies for virtual care in patients with hematologic malignancies is feasible, even in high-risk populations, and that its judicious application can serve as a lever for improving clinical outcomes and patient experiences.

Virtual care use includes app-, phone-, and video-based interventions. While no study has directly compared specific interventions, patient satisfaction has been found to be high across all methods for delivery of care. In other patient populations, there has been a preference towards telephone visits among older patients, with younger patients preferring video visits [34]. Further studies are needed in the hematologic malignancy setting in order to determine which interventions are most effective for specific patient populations.

For patients with indolent and aggressive hematologic malignancies, virtual care can be an effective method of care delivery, which can also translate to improved clinical outcomes [23]. Virtual care was also associated with high patient and provider satisfaction in several clinical settings, including patients with high-risk conditions, such as after stem cell transplant [18,19]. This high rate of patient satisfaction in patients with hematologic malignancies is similar to what has been described in the solid tumor population, where 45–66% of patients prefer telemedicine visits [13,35,36]. Avoidance of travel times, time away from work, and direct costs associated with travel have all been found to improve quality of life [37]. Younger patients with hematologic malignancies are often working through treatment [38,39,40], and virtual care may provide a particular benefit by avoiding long travel and wait times for care and allowing increased flexibility in their schedules. Further studies of patients with hematologic malignancies should assess the impact of virtual care on clinical outcomes, healthcare utilization, and specific aspects of patient satisfaction with care [7].

Virtual care for patients with hematologic malignancies also appears to have benefits across a wide range of geographic and resource settings. While most of the published literature has focused on high-resource settings, Poudyal et al. showed how app-based technologies can be used to enhance care in resource-limited settings [21]. Siddiquee et al. also found in their review that virtual care in resource-limited settings can help to overcome the barriers of shortages in healthcare providers, direct and indirect travel-related costs, and extreme environmental conditions [41]. Furthermore, virtual care in primary care and emergency medicine settings has also been shown to be cost-effective [42], emphasizing the importance of capitalizing on technology to help overcome major challenges faced in care delivery in settings where in-person visits are not possible or less valuable.

This study must be considered in the context of its strengths and limitations. Only 15 studies addressing the use of virtual care in patients with hematologic malignancies were identified, reflecting the limited literature published on this topic, and underscoring the need to benchmark the status quo in order to help advance research in this emerging and important field. Only one study included patients from a randomized trial [18], and a majority of prospective studies assessed a small number of patients for a short period of time. No studies specifically quantified the time or resources needed in order to respond to app-based messages. While the studies importantly addressed patient and provider experiences, further research is needed on system-related outcomes, including cost-saving and resource allocation. 

## 5. Conclusions

This scoping review found preliminary evidence that virtual care is feasible in patients with various hematologic malignancies, including patients after stem cell transplant. Although providers faced some challenges related to the specific technologies, high rates of patient satisfaction were reported, and there was no evidence to suggest that it negatively affected clinical outcomes. However, integrating a new modality into routine practice requires ongoing evaluation centered around the quality domains, including effectiveness, safety, timeliness, patient-centeredness, equitability, and efficiency [43]. Further research is needed on the appropriateness of virtual care for specific populations with hematologic malignancies, the specific circumstances in which it should be used, and the downstream consequences of this integration for patients, providers, and health systems.

## Supplementary Materials

The following are available online at https://www.mdpi.com/article/10.3390/curroncol29020076/s1, Methodology S1: Search strategy; Table S1: Characteristics of articles included in the scoping review.
