# Supplementary material for: The Use of Virtual Care in Patients with Hematologic Malignancies: A Scoping Review
Source: Curr Oncol. 2022 Feb 7;29(2):892–900. doi: 10.3390/curroncol29020076 (PMC8871074; doi:10.3390/curroncol29020076)
Supplement: Supplementary file 1 [file curroncol-29-00076-s001.zip › curroncol-1538504-supplementary.pdf]

*Supplementary Material*

# The Use of Virtual Care in Patients with Hematologic Malignancies – A Scoping Review

**Adam Suleman [1,*], Abi Vijenthira [2], Alejandro Berlin [3,4], Anca Prica [2] and Danielle Rodin [3,4]**

[1] Faculty of Medicine, University of Toronto
[2] Division of Medical Oncology and Hematology, Princess Margaret Cancer Centre
[3] Radiation Medicine Program, Princess Margaret Cancer Centre
[4] Department of Radiation Oncology, University of Toronto
* Correspondence: adamsuleman03@gmail.com

**Methodology S1.** Search Strategy.

***PubMed***

("Hematologic Neoplasms"[Majr]) OR "Leukemia"[Mesh]) OR "Lymphoma"[Mesh]) OR "Multiple Myeloma"[Mesh]) OR ("Hematopoietic Stem Cell Transplantation"[Majr]) OR "Stem Cell Transplantation"[Majr]) AND "Telemedicine"[Majr]

***MEDLINE***

| # | Search Statement |
|---|---|
| 1 | exp Hematologic Neoplasms |
| 2 | exp Leukemia |
| 3 | exp Lymphoma |
| 4 | exp Multiple Myeloma |
| 5 | exp Telemedicine |
| 6 | ehealth.mp. |
| 7 | mhealth.mp. |
| 8 | virtual care.mp. |
| 9 | 1 or 2 or 3 or 4 |
| 10 | 5 or 6 or 7 or 8 |
| 11 | 9 and 10 |

***EMBASE***

| # | Search Statement |
|---|---|
| 1 | exp hematologic malignancy |
| 2 | exp leukemia |
| 3 | exp lymphoma |
| 4 | exp myeloma |

5 telehealth/ or telemedicine

6 ehealth.mp.

7 mhealth.mp.

8 virtual care.mp.

9 1 or 2 or 3 or 4

10 5 or 6 or 7 or 8

11 9 and 10

***Scopus***

( TITLE-ABS-KEY ( hematologic AND malignancy ) OR TITLE-ABS-KEY ( leukemia ) OR TITLE-ABS-KEY ( lymphoma ) OR TITLE-ABS-KEY ( myeloma ) AND TITLE-ABS-KEY ( virtual AND care ) OR TITLE-ABS-KEY ( telemedicine ) OR TITLE-ABS-KEY ( ehealth ) OR TITLE-ABS-KEY ( mhealth ) )

***CINAHL***

((virtual care or telehealth or telemedicine or telemonitoring or telepractice or telenursing or telecare) OR (virtual care or mhealth or ehealth or digital health or telehealth)) AND (S2 AND S3)

**Table S1.** Characteristics of articles included in the scoping review.

| Source | Country | Design | Population | Main intervention | Main outcomes |
|---|---|---|---|---|---|
| Rueter et al. 2014 [23] | France | Retrospective cohort study | Diffuse large B-cell lymphoma on treatment with R-CHOP or R-CHOP-like chemoimmunotherapy<br>▪ 418 adult patients | *Phone-based:*<br>▪ Telemedicine phone call by a coordinating nurse to proactively ask about symptoms and side-effects of treatment | *Descriptive:*<br>▪ 143 patients (34.2%) had virtual phone-based care<br><br>*Survival outcomes:*<br>▪ Use of virtual care associated with a 40% decreased risk of death |
| Narayanan et al. 2021 [30] | United States | Retrospective case-control study | Patients with both solid tumour and hematologic malignancies<br>▪ 842 new patients 2019<br>▪ 509 new patients 2020 | *Mixed-methods of virtual care:*<br>▪ New patient integrative oncology telehealth consults<br>▪ Telehealth via phone-based or Facetime<br>▪ Zoom platform later in 2020<br><br>*Data assessment:*<br>▪ Patient questionnaire | *Descriptive:*<br>▪ 55 patients (7%) with hematologic malignancies in 2019<br>▪ 36 patients (7%) with hematologic malignancies in 2020<br><br>*Patient-related outcomes:*<br>1. For all oncology patients - decreased anxiety, depression and fatigue<br>2. For all oncology patients – decreased financial distress with the use of telehealth |

| | | | | | |
|---|---|---|---|---|---|
| Dyer et al. 2016 [26] | Australia | Retrospective survey analysis | Adult patients after allogeneic stem cell transplant<br>1. 1475 patients | *Video-based:*<br>1. Follow-up with a satellite clinic using telemedicine services<br><br>*Data assessment:*<br>2. Patient survey | *Descriptive:*<br>1. 441 patients completed the survey<br><br>*Patient satisfaction:*<br>2. 328 out of 441 patients (74%) preferred follow-up at the transplant centre or at a satellite clinic with telemedicine services<br>3. 18 out of 441 (4.1%) preferred telemedicine alone |
| Ector et al. 2020 [20] | Netherlands | Prospective pilot test | Chronic myeloid leukemia<br>▪ 11 patients<br>▪ 1 caregiver<br>▪ 13 hematologists | *App-based:*<br>▪ Website and mobile app<br><br>*Data assessment:*<br>▪ Focus groups during pilot testing | *Patient satisfaction:*<br>▪ Positive response to screen consultations, improved convenience<br><br>*Provider satisfaction:*<br>▪ Good system but concerns with technical issues |
| Poudyal et al. 2020 [21] | Nepal | Descriptive study | Acute leukemia and other hematologic malignancies | *App-based:*<br>▪ Free call and text app used for communication during the COVID-19 pandemic | *Patient-related outcomes:*<br>▪ No missed chemotherapy<br>▪ Facilitated hospital admissions |
| Condom et al. 2021 [24] | Spain | Retrospective cohort study | Adult patients with hematologic malignancies | *Phone-based:*<br>▪ Appointments transitioned from in-person to phone-based | *Descriptive:*<br>▪ Number of phone visits increased by 761% |
| Koffman et al. 2019 [27] | United States | Retrospective cohort study | Adult patients with chronic lymphocytic leukemia<br>▪ 84 patients | *Video-based:*<br>▪ Consults with experts using a video platform | *Patient-related outcomes:*<br>▪ 64% were more confident in their diagnosis<br>▪ 82% had a better understanding of their disease and treatment course |
| Koffman et al. 2020 [28] | United States | Retrospective cohort study | Adult patients with chronic lymphocytic leukemia<br>▪ 105 patients | *Video-based:*<br>▪ Consults with experts using a video platform | *Patient-related outcomes:*<br>▪ 95% of patients found the online consultation was important for their understanding<br>▪ 99% of patients would recommend a virtual specialist consult |
| Zhang et al. 2019 [22] | China | Retrospective cohort study | Patients with lymphoma after discharge from hospital | *App-based:*<br>▪ Scheduled tasks and requests for appointments through app | *Descriptive:*<br>▪ 218 patients had 616 side-effect symptom alerts triggered |

| | | | | | |
|---|---|---|---|---|---|
| | | | ▪ 856 individuals | ▪ Oncologists given access to app data and could consult remotely to manage side-effects | *Patient-related outcomes:* ▪ All patients with triggers were followed with a mobile consultation with the attending physician |
| Overend et al. 2008 [29] | Canada | Retrospective cohort study | Patients with indolent hematologic malignancies ▪ 53 patients, majority with chronic lymphocytic leukemia or follicular lymphoma | *Video-based:* ▪ Teleclinic interviews conducted by an oncology nurse, reviewed with the attending physician *Data assessment:* ▪ Patient satisfaction questionnaires | *Patient-related outcomes:* ▪ 62% preferred teleclinic visits for follow-up ▪ 20% had a neutral preference |
| Runge et al. 2020 [32] | United States | Retrospective cohort study | Adult patients with lymphoma on treatment who required dose modifications ▪ 1290 treatment cycles | *Data assessment:* ▪ Review of clinical notes of charts to determine reasoning for dose reductions | *Patient-related outcomes:* ▪ Dose adjustments were made for 144 cycles, and only 7 were based on physical exam findings alone |
| Lupo-Stanghellini et al. 2020 [25] | Italy | Retrospective cohort study | Patients after allogeneic stem cell transplant | *Phone-based:* ▪ Phone teleconsults during the initial phase of the COVID-19 pandemic | *Descriptive:* ▪ 29 out of 50 patients with chronic graft-versus-host disease were evaluated by teleconsult ▪ 125 out of 203 visits for non-respiratory related illnesses were conducted virtually |
| Fattizzo et al. 2020 [31] | Italy | Retrospective cohort study | Patients with benign and malignant hematologic conditions | *Mixed-methods of virtual care:* ▪ Phone and video-based assessments during the initial phase of COVID-19 pandemic | *Descriptive:* ▪ The number of telemedicine visits increased significantly by the middle of the first week of the pandemic, with 400-500 visits done virtually and only 200 in-person |
| Applebaum et al. 2012 [18] | United States | Randomized trial | Patients with clinical post-traumatic stress disorder after hematopoietic stem cell transplant ▪ 46 patients | *Phone-based:* ▪ Randomization to phone assessment only or phone assessment with cognitive behaviour therapy delivered over the phone for 10 sessions | *Patient-related outcomes:* ▪ General distress decreased with cognitive behavioural therapy ▪ Increased therapeutic alliance with cognitive behavioural therapy |
| Nawas et al. 2020 [19] | United States | Prospective pilot study | Patients after hematopoietic stem cell transplant | *Video-based:* ▪ Video visits conducted during hospital admission and as an outpatient | *Patient-related outcomes:* ▪ High satisfaction with virtual visits (4.12/5 rating) *Provider-related outcomes:* |

| | | |
|---|---|---|
| | ▪ 20 patients post-autologous stem cell transplant<br>▪ 5 patients post-allogeneic stem cell transplant | ▪ 63% of providers had technical problems during virtual physical examinations<br>▪ Low satisfaction with virtual visits (2.64/5 rating) |
